# Combination of Tractography, Intraoperative Computed Tomography and 5-Aminolevulinic Acid Fluorescence in Stereotactic Brain Biopsies: A Case Series

**DOI:** 10.3390/jpm14040357

**Published:** 2024-03-28

**Authors:** Edoardo Mazzucchi, Gianluca Galieri, Fabrizio Pignotti, Pierluigi Rinaldi, Giovanni Sabatino, Giuseppe La Rocca

**Affiliations:** 1 Unit of Neurosurgery, IRCCS Regina Elena National Cancer Institute, 00144 Rome, Italy; 2 Institute of Neurosurgery, IRCCS Fondazione Policlinico Universitario Agostino Gemelli, Catholic University, 00168 Rome, Italy; giancula.galieri@policlinicogemelli.it (G.G.); giovanni.sabatino@policlinicogemelli.it (G.S.); giuseppe.larocca@policlinicogemelli.it (G.L.R.); 3 Unit of Neurosurgery, Mater Olbia Hospital, 07026 Olbia, Italy; fabrizio.pignotti@materolbia.com; 4 Unit of Radiology, Mater Olbia Hospital, 07026 Olbia, Italy; pierluigi.rinaldi@materolbia.com

**Keywords:** fluorescence, glioma, image guided surgery, stereotactic biopsy, intraoperative pathology

## Abstract

Stereotactic needle biopsy (SNB) may be performed to collect tissue samples from lesions not amenable to open surgery. Integration of tractography, intraoperative imaging and fluorescence has been applied to reduce risk of complications and confirm the adequacy of bioptic specimens. Clinical and radiological data from patients who underwent stereotactic needle biopsy with the use of intraoperative CT, tractography and 5-aminolevulinic acid (5-ALA) fluorescence in a single Hospital were retrospectively reviewed to evaluate the accuracy and safety of the procedure. Seven patients were included in the study, and all the collected specimens showed red fluorescence. In six of them, the final histopathological diagnosis was grade 4 glioblastoma IDH-wt and in the other case it was Diffuse large B-Cell Lymphoma. The integration of tractography, intraoperative CT and 5-ALA as an intraoperative marker of diagnostic samples may be suggested in biopsies of suspect gliomas and lymphomas. The cost-effectiveness of the procedure should be evaluated in future studies.

## 1. Introduction

Stereotactic needle biopsy is a surgical technique frequently applied to collect diagnostic tissue from deep seated tumors [1] in order to define the nature of an abnormal signal discovered on diagnostic imaging. Two main methods have been developed over the years in order to maximize the safety, precision and reliability of this procedure, both based on pre-operative imaging (MRI, CT) [2]: the frame-based and the frameless technique. In both cases, softw allows a trajectory to the biopsy point to be planned by defining an entry point and the target point [3,4,5]. It is frequently performed in patients with poor prognosis, for tumors not amenable to resection due to factors related to the position of the lesion or to the capacity of the patients to undergo a major surgery [6]. There are two main risks of the procedure: inadequate tissue sampling and worsening of neurological function due to direct lesion or, more frequently as a consequence of bleeding [5,7,8,9]. In both cases, the patient may lose the possibility of performing an adjuvant treatment as a consequence of the worsened functional status or of the time spent on a second surgical intervention. Advancements of radiological imaging techniques allow identification of small vessels with high-definition MRI or CT angiographic sequences, thus reducing the risk of bleeding. The use of intraoperative pathological assessment, frequently with the assistance of frozen section, has also been applied in the last decades to reduce the risk of inadequate tissue sampling. In fact, before its introduction, the non-diagnostic biopsies were reported to be up to 24% in some case series [10]. The most commonly used method is Frozen Section analysis, which has shown to agree with final pathology in more than 85% of cases in CNS tumors [11].

5-Aminolevulinic acid (5-ALA) was first introduced in brain surgery by Stummer et al. in 1998 [12]. It was approved by European Medicines Agency in 2007 and Food and Drug Administration in the US in 2017 as an intra-operative optical marker for tumor visualization in patients with suspect malignant gliomas on pre-operative imaging [13].

It is an intermediate metabolite of hemoglobin metabolic pathway as it is trans-formed in Protoporphyrin IX (PPIX), a porphyrin photosensitizer, in the mitochondria [14]. Although the exact mechanism has not been clarified, a possible explanation is that tumoral cells with decreased expression of ferrochelatase (FC) and functional heme bio-synthetic enzymes preceding FC would accumulate PPIX. Another hypothesis is that tumors have a poorly-developed vascular drainage and this results in higher concentrations of PPIX [15,16].

Photodynamic diagnosis using 5-ALA was first introduced in stereotactic biopsies by Yamaguchi et al. as a rapid and effective method to confirm the presence of tumoral tissue in biopsy samples [17]. Since then, it has become a possible tool, in association with intra-operative pathology, to improve the diagnostic yield of brain biopsies with interesting results [1,18,19,20,21,22,23,24].

Intraoperative imaging has also been described in stereotactic needle biopsies by some authors to improve accuracy and safety of the procedure [25], even in combination with 5-ALA fluorescence [18,20,22,26,27,28].

Integration of intraoperative fluorescence, intraoperative imaging and advanced pre-operative imaging such as tractography of white matter fiber tracts has been proposed in neuro-oncological surgery to accomplish the so-called maximal safe tumor resection [29,30,31]. A balance between diagnostic yield and risk of the procedure should nevertheless be considered for stereotactic needle biopsy; the integration of multiple techniques can help the surgeon in planning the best trajectory and in limiting intraoperative risk without losing diagnostic accuracy.

We report our experience in a peripheral neurosurgical center with the combination of pre-operative tractography, intraoperative fluorescence with 5-aminolevulinic acid (5-ALA) and intraoperative-CT for stereotactic needle biopsy of cerebral lesion.

## 2. Materials and Methods

We performed a retrospective analysis of clinical, radiological and intraoperative data of patients who underwent frameless stereotactic needle biopsy for brain tumor in a single Neurosurgical Center. Only adult patients were included.

The following data were considered: tumor location, fluorescence-positivity, pathology and post-operative complications.

## 3. Results

Seven patients underwent frameless stereotactic needle biopsy in our hospital between 2019 and 2023 (Table 1).

The day before surgery, a contrast enhanced MRI was performed. The MRI protocol was comprised of the following sequence protocol: T1 (voxel size = 1 × 1 × 1 mm, FOV = 24 cm, slice thickness = 1 mm, TA = 3 min); T2 (voxel size = 0.8 × 0.9 × 2.6 mm, FOV = 26 cm, slice thickness = 2.6 mm, TA = 5 min); FLAIR (voxel size = 0.9 × 1.2 × 4 mm, FOV = 24, slice thickness = 4 mm, TA = 2.23 min). Diffusion images were acquired with the following settings: 32 directions; TR = 10.092 ms; b-value = 1000 s·mm^−2^; acquired voxel size = 2.1 mm isotropic; field of view: 240 mm; no slice gap; 26 slices; and scan time 6 min 11 s.

They received 20 mg/kg of 5-ALA (Gliolan^®^, Medac, Wedel, Germany) orally 5 h before anesthesia induction. An intra-operative CT scan was performed in all cases. With the aid of the neuronavigation system (BrainLab AG, Munchen, Germany), tractography of the cortico-spinal tract was performed and fused with anatomical images for surgical planning. In particular, the entry point, target and trajectory were planned in order to reach the enhancing portion of the lesion while avoiding any vessel, sulcus, the ependyma and the cortico-spinal tract fibers.

**Figure 1:**
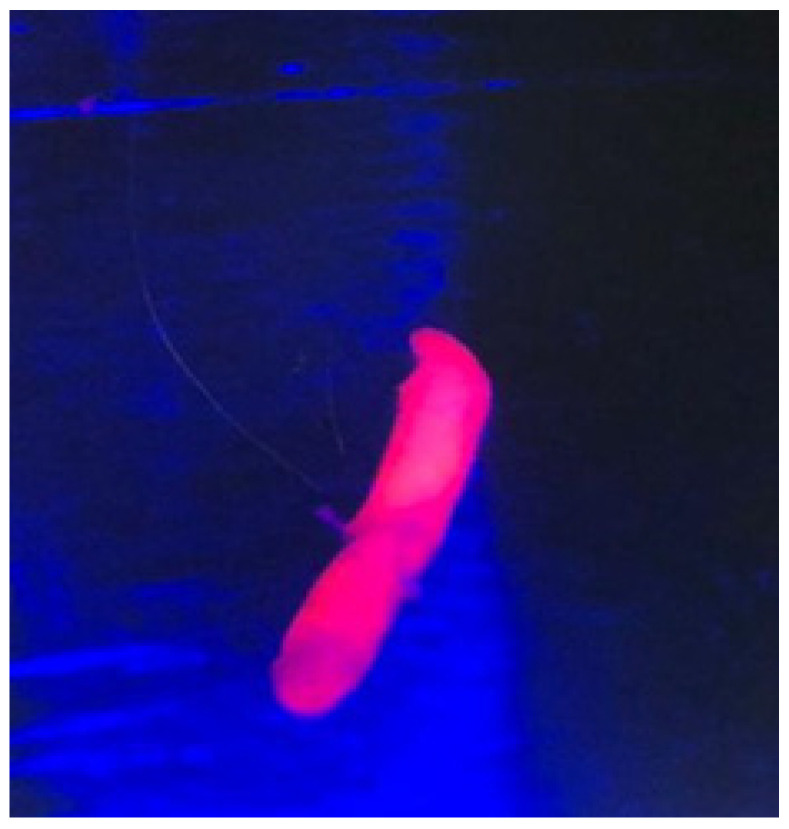
Visualization of the red fluorescent sample under the light of the microscope to confirm the adequacy of the tissue.

**Figure 2:**
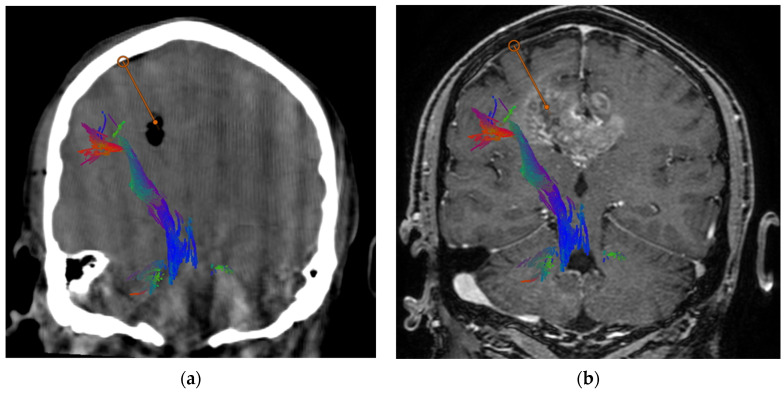
Intraoperative CT scan (**a**) and pre-operative MRI (**b**) coronal images. Intraoperative CT shows the presence of air bubble at the planned target. Moreover, it rules out immediate bleeding. The red line represents the planned trajectory between the entry point and the target. Corticospinal tract is also displayed to confirm it has been avoided by the trajectory.

After a linear skin incision, a burr hole was drilled at the planned entry point. The dura mater was coagulated and then opened with an X-shaped incision. The navigated biopsy needle was then inserted at the entry point and gently pushed deep down along the planned trajectory to the target site with a dedicated articulated arm (VarioGuide, BrainLab AG, Germany). Tissue samples were collected between 5 mm more superficial and 5 mm deeper than the target point with different orientations of the needle. The needle was then carefully extracted and the specimens were tested for fluorescence under the light of the surgical microscope (Leica Microsystems M530, Wetzlar, Germany). If the tissue sample showed fluorescence (Figure 1), no additional samples were taken, and the specimen was sent for final pathology. If no fluorescence was found, additional samples were collected. In no case was a second trajectory necessary as a positive sample was found in each case. Intra-operative pathology was performed only in one case. In all cases, an intra-operative CT (iCT) scan was performed after the end of surgery in order to exclude acute complications and to confirm the reaching of the target also from a radiological point of view (presence of air bubble at the target) (Figure 2). To protect patients from photosensitivity, they were kept in a dimly lit room 24 h after the procedure.

No complications from the use of 5-ALA or post-operative bleedings were reported. One patient reported a slight transient worsening of the pre-existing motor impairment of his left arm.

In all patients, at least one of the specimens collected during stereotactic needle biopsy showed red fluorescence. In six of them, the final histopathological diagnosis was GBM IDH-wt (grade IV, WHO 2021), while in one case it was Diffuse large B-Cell Lymphoma. In the only case in which intra-operative frozen section was conducted (Patient 2), the intraoperative pathological analysis suggested the diagnosis of malignant glioma, which was then confirmed by the final pathological diagnosis.

## 4. Discussion

Stereotactic needle biopsy is a procedure commonly used to collect tissue samples from lesions that are considered not amenable to resection and are difficult to reach with open surgery. It may be performed with a stereotactic frame or, more recently, without frame, the so-called frameless technique. In both techniques, the planning of the entry point, trajectory and target is based on pre-operative imaging (usually MRI with contrast). The surgeon must plan a trajectory avoiding vessels, sulci (in which small vessels may be encountered) and, preferably, ventricles. The target should be the most representative of the pathology. For example, in suspect high-grade gliomas, the enhancing portion of the tumor is the preferred target, while the central necrotic area is not useful to obtain diagnostic tissue.

The procedure is considered minimally invasive, but it carries potential complications, such as hemorrhage, worsening of brain oedema, infection and seizures. The collection of non-diagnostic material is another potential risk. In case of inadequate sampling, the treatment must be delayed and a second surgery must be planned with adjunctive risks related to surgery. Moreover, the time elapsed without therapy in these frail patients may imply progression of the tumor and potentially loss of the opportunity to treat the patient at all. In order to reduce the probability of inadequate sampling, intraoperative pathology, usually with frozen section, has frequently been added to the procedure [32]. This additional step of the procedure requires facilities and, most importantly, the presence of an experienced neuropathologist. As a matter of fact, intraoperative pathology should provide a clear indication to the surgeon in a short window of time and with limited diagnostic tools. In a small peripheral neurosurgical center, where the volume of neuro-oncological patients does not allow sufficient experience in this field of pathology, it is very difficult to have access to a specialized neuropathologist. In our specific hospital, a neuropathologist was not available; in the single case in which intraoperative pathology was performed, we involved a consultant from another hospital with considerable logistical and organizational effort. The planning of a biopsy with the presence of an expert neuropathologist may imply significant additional delay in the planning of surgery, so we do not consider this strictly necessary when the radiological suspect is highly indicative of high-grade glioma. On the other hand, previous papers suggest that intraoperative pathology is not necessary for enhancing lesions (in which radiological data suggest high-grade glioma or lymphoma) when 5-ALA fluorescence was positive [23,24,33], as it would prolong surgical time (and theoretically lead to more complications) without significant modification of the diagnostic accuracy of the biopsy. Moreover, in our case series, the combination with an iCT scan provides further evidence that the target has been reached. It is a double control, but in our opinion, it is not redundant. As a matter of fact, the iCT confirms the anatomic location of the target [25], while the fluorescence gives us information about metabolic activity of the collected tissue. It is frequent, for example, even in our small experience, that the observation of different fluorescence gradation, from a pale to a bright red, in different samples and the samples collected at the planned target (usually the periphery of the enhancing component of the tumor) are not necessarily the most fluorescent ones. We could then affirm that 5-ALA fluorescence and iCT are complementary techniques in the proposed workflow.

In our opinion, additional trajectories must be avoided when the fluorescence is clearly positive in order to reduce the risk of bleeding. In parallel with the well-known concept of “maximal safe resection”, we could define this as a “minimal safe biopsy”. In our and in previous case series, positive fluorescence guaranteed a high diagnostic yield so any additional sampling would not be justified.

The safety of the procedure is also improved by integrating pre-operative tractography in the planning of trajectory, thus reducing the theoretical risk of damage to relevant fiber bundles like the corticospinal tract (Figure 2). A larger patient sample should be used to corroborate our impression that the recognition of the cortico-spinal tract (CST) may have a clinical impact in reducing the risk of motor impairment. A small hemorrhage may provoke a motor impairment, or it may be pauci-symptomatic; this may be a consequence of the distance of the needle trajectory from CST. The inclusion of tractography in the pre-surgical imaging protocol and the integration of the position of the CST among the various factors to be considered in the planning could be, in our opinion, a valuable help in some cases. The described case series is too small, and we have not encountered any hemorrhage, so we cannot test this hypothesis in the present study.

Operation time is also a relevant issue, related to complication, especially in this population, which includes patients with high surgical risk [21]. Two papers [21,33] reported a statistically significant reduction both in the length of surgery and in the number of biopsy samples for fluorescent cases.

Operation time is also a substantial factor for another important element to be evaluated: the cost-benefit ratio. According to the wide variability of organizational context, it is very difficult to provide evidence-based results in this specific field. A computation of the cost of the administrated drug, of a neuronavigation system integrating an iCT scanner and of the MRI scan including sequences suitable for tractography would be very difficult due to the high variability in different healthcare system. For instance, an evaluation of the cost of the procedure has been performed by Millesi et al.; the Authors declare a cost of EUR 65 for 5-ALA and EUR 1100 for frozen section for each patient; they conclude that to perform frozen section only in non-fluorescent cases is a cost-efficient protocol. However, the cost of the drug is much higher in other countries (see for example [34]). At the same time, the price of frozen section may greatly vary; the organization of team and facilities for frozen section and the availability of an expert neuropathologist is very difficult in some peripheral hospitals, and many of the patients who require a stereotactic biopsy may not be able to travel to a higher-volume neurosurgical center. According to previous literature, 5-ALA fluorescence can be considered a reliable marker for diagnostic tissue in bioptic samples in cases of suspect 5-ALA-capturing tumors at pre-operative imaging. This would avoid unnecessary extension of the duration of surgery and additional sampling, both theoretically linked to higher risk of complications. On the other hand, the iCT scan implies some more time spent in the surgical theatre (in our experience 9–15 min in total), which nevertheless, in our experience, does not offset the time saved by avoiding waiting for intraoperative pathologic diagnosis.

In case of non-enhancing lesions, intraoperative pathologic diagnosis with frozen section may still be necessary to reduce the risk of inadequate sampling. Non-enhancing lesions rarely need a stereotactic biopsy; the underlying lesion is usually less aggressive, and a slower progression is expected. In these cases, some delay of the intervention in order to organize an intraoperative pathologic diagnosis is justified and does not imply additional risk for the patient.

An evident limitation of the present study is the limited number of patients and the absence of non-enhancing lesions in the case series. Future studies are needed to confirm our findings and to provide more accurate indications for clinical practice.

## 5. Conclusions

Integration of 5-ALA and iCT is a valuable approach in stereotactic biopsy to confirm the adequacy of the sampling for suspect high-grade gliomas on pre-operative imaging. Further studies are necessary to evaluate the diagnostic yield, safety and cost-effectiveness of the procedure in a larger population.

## Figures and Tables

**Table 1:** Clinical and pathological data of the patients included in the case series. Abbreviations: ALA: aminolevulinic acid; CC: corpus callosum; IDH: isocitrate dehydrogenase.

Patient ID	Tumor Location	5-ALA Fluorescence	Intra-Operative Pathology	Final Pathology	Complication
1	Right parietal and CC	Yes	No	Glioblastoma IDH-wt	None
2	Right parietal	Yes	Yes (malignant glioma)	Glioblastoma IDH-wt	Worsening of left arm motor function
3	Bilateral frontal and CC	Yes	No	Glioblastoma IDH-wt	None
4	Bilateral frontal and CC	Yes	No	DLBCL	None
5	Bilateral frontal and CC	Yes	No	Glioblastoma IDH-wt	None
6	Left temporal and insular	Yes	No	Glioblastoma IDH-wt	None
7	Bilateral parietal and CC	Yes	No	Glioblastoma IDH-wt	None

## Data Availability

The data presented in this study are available on request from the corresponding author.

## References

[1] Bex A., Mathon B. (2022). Advances, Technological Innovations, and Future Prospects in Stereotactic Brain Biopsies. Neurosurg. Rev..

[2] Dhawan S., He Y., Bartek J., Alattar A.A., Chen C.C. (2019). Comparison of Frame-Based Versus Frameless Intracranial Stereotactic Biopsy: Systematic Review and Meta-Analysis. World Neurosurg..

[3] Kesserwan M.A., Shakil H., Lannon M., McGinn R., Banfield L., Nath S., Alotaibi M., Kasper E., Sharma S. (2021). Frame-Based versus Frameless Stereotactic Brain Biopsies: A Systematic Review and Meta-Analysis. Surg. Neurol. Int..

[4] Woodworth G.F., McGirt M.J., Samdani A., Garonzik I., Olivi A., Weingart J.D. (2006). Frameless Image-Guided Stereotactic Brain Biopsy Procedure: Diagnostic Yield, Surgical Morbidity, and Comparison with the Frame-Based Technique. J. Neurosurg..

[5] Riche M., Amelot A., Peyre M., Capelle L., Carpentier A., Mathon B. (2021). Complications after Frame-Based Stereotactic Brain Biopsy: A Systematic Review. Neurosurg. Rev..

[6] Balducci M., Chiesa S., Diletto B., D’Agostino G.R., Mangiola A., Manfrida S., Mantini G., Albanese A., Fiorentino A., Frascino V. (2012). Low-Dose Fractionated Radiotherapy and Concomitant Chemotherapy in Glioblastoma Multiforme with Poor Prognosis: A Feasibility Study. Neuro Oncol..

[7] Mizobuchi Y., Nakajima K., Fujihara T., Matsuzaki K., Mure H., Nagahiro S., Takagi Y. (2019). The Risk of Hemorrhage in Stereotactic Biopsy for Brain Tumors. J. Med. Investig..

[8] He Z., Zhu C.X.L., Chan D.T.M., Cheung T.C.Y., Ng H.-K., Mok V.C.T., Poon W.S. (2024). Diagnostic Accuracy and Field for Improvement of Frameless Stereotactic Brain Biopsy: A Focus on Nondiagnostic Cases. J. Neurol. Surg. A Cent. Eur. Neurosurg..

[9] Riche M., Marijon P., Amelot A., Bielle F., Mokhtari K., de Chambrun M.P., Joncour A.L., Idbaih A., Touat M., Do C.-H. (2022). Severity, Timeline, and Management of Complications after Stereotactic Brain Biopsy. J. Neurosurg..

[10] Zoeller G.K., Benveniste R.J., Landy H., Morcos J.J., Jagid J. (2009). Outcomes and Management Strategies after Nondiagnostic Stereotactic Biopsies of Brain Lesions. Stereotact. Funct. Neurosurg..

[11] Regragui A., Amarti Riffi A., Maher M., El Khamlichi A., Saidi A. (2003). Accuracy of intraoperative diagnosis in central nervous system tumors: Report of 1315 cases. Neurochirurgie.

[12] Walter S., Susanne S., Simon W., Herbert S., Clemens F., Claudia G., Alwin E.G., Rainer K., Hans J.R. (1998). Intraoperative Detection of Malignant Gliomas by 5-Aminolevulinic Acid-Induced Porphyrin Fluorescence. Neurosurgery.

[13] Hadjipanayis C.G., Stummer W. (2019). 5-ALA and FDA Approval for Glioma Surgery. J. Neurooncol..

[14] Mahmoudi K., Garvey K.L., Bouras A., Cramer G., Stepp H., Jesu Raj J.G., Bozec D., Busch T.M., Hadjipanayis C.G. (2019). 5-Aminolevulinic Acid Photodynamic Therapy for the Treatment of High-Grade Gliomas. J. Neurooncol..

[15] Gibson S.L., Nguyen M.L., Havens J.J., Barbarin A., Hilf R. (1999). Relationship of δ-Aminolevulinic Acid-Induced Protoporphyrin IX Levels to Mitochondrial Content in Neoplastic Cells in Vitro. Biochem. Biophys. Res. Commun..

[16] Kaneko S., Eljamel M.S. (2017). Fluorescence Image-Guided Neurosurgery. Future Oncol..

[17] Yamaguchi F., Takahashi H., Teramoto A. (2007). Photodiagnosis for Frameless Stereotactic Biopsy of Brain Tumor. Photodiagnosis Photodyn. Ther..

[18] Malinova V., von Eckardstein K., Mielke D., Rohde V. (2020). Diagnostic Yield of Fluorescence-Assisted Frame-Based Stereotactic Biopsies of Intracerebral Lesions in Comparison with Frozen-Section Analysis. J. Neurooncol..

[19] Kiesel B., Wadiura L.I., Mischkulnig M., Makolli J., Sperl V., Borkovec M., Freund J., Lang A., Millesi M., Berghoff A.S. (2021). Efficacy, Outcome, and Safety of Elderly Patients with Glioblastoma in the 5-ALA Era: Single Center Experience of More Than 10 Years. Cancers.

[20] Marbacher S., Klinger E., Schwyzer L., Fischer I., Nevzati E., Diepers M., Roelcke U., Fathi A.-R., Coluccia D., Fandino J. (2014). Use of Fluorescence to Guide Resection or Biopsy of Primary Brain Tumors and Brain Metastases. Neurosurg. Focus.

[21] Shofty B., Richetta C., Haim O., Kashanian A., Gurevich A., Grossman R. (2019). 5-ALA-Assisted Stereotactic Brain Tumor Biopsy Improve Diagnostic Yield. Eur. J. Surg. Oncol..

[22] von Campe G., Moschopulos M., Hefti M. (2012). 5-Aminolevulinic Acid-Induced Protoporphyrin IX Fluorescence as Immediate Intraoperative Indicator to Improve the Safety of Malignant or High-Grade Brain Tumor Diagnosis in Frameless Stereotactic Biopsies. Acta Neurochir..

[23] Widhalm G., Minchev G., Woehrer A., Preusser M., Kiesel B., Furtner J., Mert A., Di Ieva A., Tomanek B., Prayer D. (2012). Strong 5-Aminolevulinic Acid-Induced Fluorescence Is a Novel Intraoperative Marker for Representative Tissue Samples in Stereotactic Brain Tumor Biopsies. Neurosurg. Rev..

[24] Yamamoto T., Ishikawa E., Miki S., Sakamoto N., Zaboronok A., Matsuda M., Akutsu H., Nakai K., Tsuruta W., Matsumura A. (2015). Photodynamic Diagnosis Using 5-Aminolevulinic Acid in 41 Biopsies for Primary Central Nervous System Lymphoma. Photochem. Photobiol..

[25] Ikeda N., Katayama Y., Kawabata S., Furuse M., Tsuji Y., Nonoguchi N., Yagi R., Kameda M., Takami T., Kuroiwa T. (2022). Frameless Stereotactic Biopsy with Intraoperative Computed Tomography “Assessment of Efficacy and Real Target Registration Error”. Neurol. Med. Chir..

[26] Hefti M., von Campe G., Moschopulos M., Siegner A., Looser H., Landolt H. (2008). 5-Aminolevulinic Acid Induced Protoporphyrin IX Fluorescence in High-Grade Glioma Surgery: A One-Year Experience at a Single Institutuion. Swiss Med. Wkly..

[27] Moriuchi S., Yamada K., Dehara M., Teramoto Y., Soda T., Imakita M., Taneda M. (2011). Use of 5-Aminolevulinic Acid for the Confirmation of Deep-Seated Brain Tumors during Stereotactic Biopsy: Report of 2 Cases. J. Neurosurg..

[28] Giordano M., Gallieni M., Zaed I., Samii A. (2019). Use of Frameless Stereotactic Navigation System Combined with Intraoperative Magnetic Resonance Imaging and 5-Aminolevulinic Acid. World Neurosurg..

[29] Mazzucchi E., La Rocca G., Ius T., Sabatino G., Della Pepa G.M. (2020). Multimodality Imaging Techniques to Assist Surgery in Low-Grade Gliomas. World Neurosurg..

[30] Mazzucchi E., La Rocca G., Hiepe P., Pignotti F., Galieri G., Policicchio D., Boccaletti R., Rinaldi P., Gaudino S., Ius T. (2022). Intraoperative Integration of Multimodal Imaging to Improve Neuronavigation: A Technical Note. World Neurosurg..

[31] Della Pepa G.M., Ius T., La Rocca G., Gaudino S., Isola M., Pignotti F., Rapisarda A., Mazzucchi E., Giordano C., Dragonetti V. (2020). 5-Aminolevulinic Acid and Contrast-Enhanced Ultrasound: The Combination of the Two Techniques to Optimize the Extent of Resection in Glioblastoma Surgery. Neurosurgery.

[32] Colbassani H.J., Nishio S., Sweeney K.M., Bakay R.A., Takei Y. (1988). CT-Assisted Stereotactic Brain Biopsy: Value of Intraoperative Frozen Section Diagnosis. J. Neurol. Neurosurg. Psychiatry.

[33] Millesi M., Kiesel B., Wöhrer A., Mercea P.A., Bissolo M., Roetzer T., Wolfsberger S., Furtner J., Knosp E., Widhalm G. (2020). Is Intraoperative Pathology Needed If 5-Aminolevulinic-Acid-Induced Tissue Fluorescence Is Found in Stereotactic Brain Tumor Biopsy?. Neurosurgery.

[34] Slof J., Díez Valle R., Galván J. (2015). Cost-Effectiveness of 5-Aminolevulinic Acid-Induced Fluorescence in Malignant Glioma Surgery. Neurología.

